# Anxiety Symptoms Before and After Single-Incision Mini-Sling Surgery in Women With Stress Urinary Incontinence: A Prospective Study

**DOI:** 10.7759/cureus.96658

**Published:** 2025-11-12

**Authors:** Drivakou Despoina, Theodoulidis Iakovos, Roussos Nikolaos, Tsiapakidou Sofia, Pantazis Konstadinos, Dinas Konstantinos, Mikos Themistoklis, Mary H Kosmidis

**Affiliations:** 1 Urogynecology Unit, 2nd Department of Obstetrics and Gynecology, Aristotle University of Thessaloniki, Hippokration General Hospital, Thessaloniki, GRC; 2 Urogynecology Unit, 1st Department of Obstetrics and Gynecology, Papageorgiou Hospital, Thessaloniki, GRC; 3 Urogynecology Unit, 1st Department of Obstetrics and Gynecology, Aristotle University of Thessaloniki, Papageorgiou General Hospital, Thessaloniki, GRC; 4 School of Psychology, Aristotle University of Thessaloniki, Thessaloniki, GRC

**Keywords:** anxiety, depression prevention, health related-quality of life, psychological wellbeing, single-incision mini-sling, state-trait anxiety inventory, stress urinary incontinence, urinary stress incontinence, woman’s mental health

## Abstract

Objective

To evaluate the impact of single-incision mini-sling (SIMS) surgery on anxiety symptoms in women with moderate to severe stress urinary incontinence (SUI), using the State-Trait Anxiety Inventory (STAI).

Methodology

A prospective cohort study was conducted in a tertiary urogynecology center (December 2022-January 2023). Forty-three women with SUI or mixed urinary incontinence (MUI; co-occurrence of stress and urgency symptoms) undergoing SIMS were assessed preoperatively and at three months postoperatively using STAI, International Consultation on Incontinence Questionnaire (ICIQ), Female Lower Urinary Tract Symptoms (FLUTS), and Patient Global Impression of Improvement (PGI-I)/Patient Global Impression of Severity (PGI-S). Women with prior incontinence surgery or pelvic organ prolapse were excluded; current psychotropic medication use with anxiolytic/antidepressant effects (e.g., Selective Serotonin Reuptake Inhibitors (SSRIs), benzodiazepines, Serotonin-Norepinephrine Reuptake Inhibitors/Tricyclic Antidepressants (SNRIs/TCAs), buspirone, atypical antipsychotics, mood stabilizers) was also excluded. STAI-State and STAI-Trait were primary outcomes; total STAI was secondary.

Results

Postoperatively, 40 (93%) participants achieved continence (negative cough stress test). Mean total STAI decreased from 50.6 ± 8.3 to 45.8 ± 7.3 (*P* < 0.001). A greater proportion of women with moderate SUI achieved normal STAI (<41) versus severe SUI (*P* = 0.011). Anxiety reduction did not differ between SUI and MUI, although MUI reported slightly lower satisfaction.

Conclusions

SIMS was associated with high continence rates and significant reductions in anxiety at three months. The findings highlight the psychological burden of incontinence and support integrating mental health assessments into urogynecologic care. Specifically, we recommend preoperative STAI screening to flag high trait anxiety, offering perioperative counseling/referral when indicated, and routine tracking of STAI/ICIQ as patient-reported outcome measures.

## Introduction

Stress urinary incontinence (SUI) is a common urological disorder among women and represents a significant public health issue. According to the International Continence Society, it is defined as the involuntary loss of urine during physical exertion, sneezing, or coughing. SUI constitutes a subtype of urinary incontinence that disproportionately affects women due to anatomical, hormonal, and obstetric factors [1]. Its global prevalence is estimated to range from 20% to 35% among adult women, with higher rates observed in individuals over 50 years of age and those with a history of multiple childbirths [2,3]. Although not life-threatening, SUI profoundly affects quality of life (QoL), contributing to emotional, physical, sexual, and occupational challenges [4].

Beyond the physical manifestation of urinary leakage, the burden of SUI encompasses substantial psychological and emotional dimensions, which are often underestimated in routine clinical care. Affected women frequently report experiences of embarrassment, diminished self-worth, frustration, and shame. These emotional consequences may lead to avoidance of physical activity, social isolation, and the deterioration of intimate relationships [5,6]. The unpredictable nature of leakage episodes often induces a persistent fear of public accidents, heightening anxiety and, in some instances, leading to clinically significant depressive symptoms [7].

An increasing body of evidence supports a strong association between SUI and mental health disorders, particularly anxiety and depression. Cross-sectional and longitudinal studies have highlighted a bidirectional relationship, whereby urinary symptoms can provoke psychological distress, while pre-existing psychological vulnerabilities may amplify symptom perception [8,9]. Many women with SUI develop heightened awareness and concern over bladder control, especially in unfamiliar or public settings. In certain populations, the prevalence of anxiety or depressive symptoms among women with moderate to severe SUI exceeds 40% [10]. Such psychiatric comorbidities not only compound the disease burden but also impact treatment satisfaction and long-term outcomes.

Optimal management of SUI should therefore extend beyond alleviating physical symptoms to also encompass psychosocial well-being. Conservative measures, such as pelvic floor muscle training (PFMT), behavioral modifications, and bladder retraining, are considered first-line therapies and may suffice in mild cases. There is a paucity of data on outcomes according to the preoperative severity of SUI [11]. However, the success rates of incontinence procedures largely depend on preoperative severity and are significantly lower in cases of more severe SUI [12]. Therefore, many women with moderate to severe symptoms ultimately require surgical intervention [13]. Mid-urethral slings (MUS) are widely regarded as the surgical gold standard, offering a favorable balance between effectiveness and safety. These slings function by providing support to the mid-urethra, thus restoring continence during increases in intra-abdominal pressure [14]. Single-incision mini-slings (SIMS) provide a minimally invasive MUS option with comparable efficacy in appropriately selected patients.

From a patient-centered perspective, outcomes such as anxiety, emotional distress, and overall well-being should be considered integral to evaluating the efficacy of SUI treatments. Anxiety, in particular, may significantly influence both pre- and postoperative experiences, shaping perceptions of success, adherence to care recommendations, and QoL. Although clinical observations suggest that regaining continence can alleviate anxiety linked to leakage, few studies have rigorously assessed this effect using standardized, validated instruments. The omission of psychological outcomes in surgical research represents a notable gap in the current literature and clinical practice.

One established tool for assessing anxiety is the State-Trait Anxiety Inventory (STAI), a psychometric instrument that distinguishes between temporary, situation-specific anxiety (state anxiety) and a more generalized predisposition toward anxiety (trait anxiety) [15]. This measure has been extensively validated in clinical and surgical populations and is particularly useful for tracking anxiety over time. We used the Greek-validated version of STAI-Y, which has demonstrated acceptable psychometric properties in Greek populations [16]. Its use in the context of SUI surgery enables a nuanced understanding of both immediate emotional responses and enduring psychological profiles of patients undergoing treatment.

Assessing the psychological impact of surgical intervention may also facilitate more personalized preoperative counseling and postoperative support. Patients with elevated trait anxiety may benefit from additional psychological interventions, regardless of surgical success. Conversely, those whose anxiety is primarily reactive to incontinence episodes may experience significant relief following restoration of continence. Recognizing these distinctions is essential for designing comprehensive, individualized care strategies that address both physical and emotional health.

On a broader level, demonstrating improvements in mental health outcomes post-surgery may have implications for health policy and service provision. Evidence supporting enhanced psychological well-being after continence surgery could inform funding decisions and increase accessibility, particularly in publicly funded healthcare systems. Moreover, incorporating mental health metrics into surgical outcome research aligns with evolving international recommendations that advocate for the use of patient-reported outcome measures (PROMs) alongside traditional anatomical or functional indicators of success [17].

In conclusion, SUI is not merely a condition of bladder dysfunction-it carries substantial psychological consequences that can profoundly affect women’s lives. While procedures such as SIMS provide promising physical results, their influence on emotional well-being remains insufficiently explored. Prospective studies employing validated psychological assessment tools are essential to advance a more holistic, patient-centered understanding of treatment success in SUI. Addressing this gap will enhance both clinical care and research, ultimately improving outcomes for women affected by this pervasive condition. In addition, the impact of pre-operative severity on postoperative psychological recovery remains underexplored and warrants stratified evaluation.

Objective

To evaluate changes in STAI-Y from preoperative assessment to three months after SIMS in women with moderate to severe SUI and SUI-predominant MUI, with the a priori hypothesis that state anxiety would show a greater reduction than trait anxiety.

## Materials and methods

From December 2022 to January 2023, we conducted a prospective cohort study in the Urogynecologic Unit of Papageorgiou General Hospital, Thessaloniki, Greece, approved by the institutional Ethics and Research Committee (approval ID: 2021-B2015-132, date: May 17, 2021). Consecutive adult women with SUI or MUI with stress-predominant symptoms (≥70% stress-predominant complaints) who were scheduled to receive SIMS surgery were enrolled. All patients were initially evaluated in the outpatient department (history, Pelvic Organ Prolapse Quantification (POP-Q), cough stress test). Exclusion criteria were prior incontinence surgery, clinically significant pelvic organ prolapse, and current use of psychotropic medications with anxiolytic or antidepressant effects (e.g., SSRIs, benzodiazepines, Serotonin-Norepinephrine Reuptake Inhibitors/Tricyclic Antidepressants (SNRIs/TCAs), buspirone, atypical antipsychotics, and mood stabilizers). All participants provided written informed consent.

Preoperative evaluation included urodynamics, cough stress testing in both supine and standing positions with a bladder fill of 200-300 mL or at maximum cystometric capacity (MCC), and introital ultrasonography to assess urethral mobility. Patient-reported outcomes included the International Consultation on Incontinence Questionnaire-Urinary Incontinence Short Form (ICIQ-UI SF) [18], the International Consultation on Incontinence Questionnaire-Female Lower Urinary Tract Symptoms (ICIQ-FLUTS) [19], and the International Consultation on Incontinence Questionnaire-Vaginal Symptoms (ICIQ-VS) [20]. Anxiety was assessed using the Greek-validated STAI-Y [16], with the State and Trait subscales administered and analyzed separately. Normal anxiety was defined as STAI scores <41 [17,21]. The primary outcomes were STAI-State and STAI-Trait, while total STAI served as a secondary outcome. STAI and other questionnaires were administered preoperatively and at three months postoperatively, a time point chosen because continence and PROMs after MUS/SIMS typically stabilize by ~8 to 12 weeks.

Intra- and postoperative variables included operative duration, intraoperative pain (visual analog scale 0-10), immediate postoperative complications, and overall satisfaction. At three months, clinical assessment comprised stress test, erosion check, POP-Q, perineal floor ultrasound (including sling mobility), and questionnaires (Patient Global Impression of Improvement (PGI-I), Patient Global Impression of Severity (PGI-S) [21], ICIQ-UI SF [18], ICIQ-FLUTS [19], ICIQ-VS [20], STAI [17]). Urethral and sling mobility were measured following a study by Schaer et al. [22]. Sexual function was also assessed using the Female Sexual Function Index (FSFI) [23]. SUI severity was classified by ICIQ-UI SF thresholds: moderate 6-12, severe ≥13 [18]. Objective continence was defined as a negative cough stress test.

Statistical analysis

Analyses were performed in JASP 0.19.1 for Windows (JASP Team, University of Amsterdam, Amsterdam, The Netherlands, 2024). Continuous variables are reported as mean ± standard deviation (SD). Pre-/post-comparisons used paired t-tests; between-group comparisons used independent-samples t-tests. Normality of change scores was assessed using the Shapiro-Wilk test. A two-tailed significance level of α = 0.05 was applied. Exact *P*-values are reported where available; values <0.001 reflect software precision. Effect sizes (Cohen’s d/dz) with 95% confidence intervals are reported for primary outcomes. Missing data were handled using listwise deletion, with no imputation. A post hoc power analysis (*d* = 0.5, α = 0.05, paired design) indicated 1 - β = 0.84 for detecting a 5-point change in STAI [24].

## Results

In total, 43 women with SUI or SUI-predominant MUI (≥70% stress-predominant complaints) were enrolled. Baseline demographics, prolapse findings, questionnaires, and urodynamic parameters are shown in Table 1. The mean age was 64.6 ± 12.0 years. Based on ICIQ-UI SF thresholds, 11 had moderate SUI (mean ICIQ-UI SF 9.9 ± 1.6) and 32 had severe SUI (mean ICIQ-UI SF 15.3 ± 1.5).

**Table 1:** Baseline characteristics. Cohort includes SUI-predominant MUI (≥70%). Severity thresholds: ICIQ-UI SF moderate 6-12; severe ≥13 [18]. *P*-values from independent-samples t-tests. Note: Bold values denote statistical significance at *P* < 0.05. MUI, mixed urinary incontinence; POP-Q, Pelvic Organ Prolapse Quantification; BMI, body mass index; BNDW, bladder neck descent (weight-bearing); BN Mob, bladder neck mobility; VLPP, Valsalva leak point pressure; ICIQ-UI SF, International Consultation on Incontinence Questionnaire-Urinary Incontinence Short Form; ICIQ-VS, International Consultation on Incontinence Questionnaire-Vaginal Symptoms; FLUTS, Female Lower Urinary Tract Symptoms; STAI, State-Trait Anxiety Inventory

Baseline characteristics	All patients (43)	SUI (33)	MUI (10)	P
Age (years)	64.60 ± 12.02	63.39 ± 11.20	68.60 ± 14.13	0.235
BMI	29.95 ± 5.43	29.24 ± 4.67	32.29 ± 7.21	0.120
Parity	2.58 ± 0.98	2.67 ± 1.02	2.30 ± 0.82	0.306
BNDW	3561 ± 719	3595 ± 527	3449 ± 1187	0.579
POP-Q Ba	-2.07 ± 0.83	-2.09 ± 0.80	-2.00 ± 0.94	0.765
POP-Q C	-6.19 ± 1.22	-6.27 ± 1.30	-5.90 ± 0.88	0.404
POP-Q GH	3.21 ± 0.64	3.15 ± 0.62	3.40 ± 0.70	0.286
POP-Q TVL	10.83 ± 1.27	10.85 ± 1.37	10.80 ± 0.91	0.917
POP-Q Bp	-1.95 ± 0.53	-2.00 ± 0.56	-1.80 ± 0.42	0.304
Pre-Op BN Mob	1.27 ± 0.71	1.33 ± 0.71	1.10 ± 0.72	0.389
Pre-Op ICIQ-UI SF Total	13.95 ± 2.84	13.85 ± 3.00	14.30 ± 2.54	0.666
Pre-Op FLUTS Total	18.84 ± 5.66	17.85 ± 5.56	22.10 ± 4.89	0.036
Pre-Op FLUTS Filling	6.09 ± 3.87	5.54 ± 3.93	7.90 ± 3.21	0.092
Pre-Op FLUTS Voiding	1.67 ± 1.71	1.48 ± 1.73	2.30 ± 1.57	0.191
Pre-Op FLUTS Incontinence	11.49 ± 3.08	11.36 ± 3.08	11.90 ± 3.21	0.635
Pre-Op ICIQ-VS Total	4.81 ± 4.53	5.00 ± 4.59	4.20 ± 4.54	0.631
VLPP	61.94 ± 32.90	64.30 ± 35.81	51.67 ± 12.09	0.405
Pre-Op STAI Total	50.65 ± 8.29	50.48 ± 8.22	51.20 ± 8.95	0.814

Postoperatively, 40 participants (93%) had a negative cough stress test; 3 (7.0%) had mild leakage on the 1-3-5 cough stress test. PGI-I and PGI-S were 1.4 ± 0.8 and 1.2 ± 0.6, respectively. ICIQ-UI SF improved from 13.9 ± 2.8 to 2.8 ± 3.4 (*P *< 0.001). FLUTS total, filling, and incontinence domains improved significantly (Table 2).

**Table 2:** Preoperative vs. postoperative results. Paired t-tests; two-tailed α = 0.05. Exact *P*-values reported where available; *P*-values <0.001 reflect software precision. Note: Bold values denote statistical significance at *P* < 0.05. BN Mob, bladder neck mobility; ICIQ-UI SF, International Consultation on Incontinence Questionnaire-Urinary Incontinence Short Form; FLUTS, Female Lower Urinary Tract Symptoms; STAI, State-Trait Anxiety Inventory

Measure	Pre-Op	Post-Op	P-value
BN Mob	1.27 ± 0.71	1.05 ± 0.50	0.118
ICIQ-UI SF Total	13.95 ± 2.84	2.84 ± 3.40	<0.001
FLUTS Total	18.84 ± 5.66	6.70 ± 5.32	<0.001
FLUTS Filling	6.09 ± 3.87	2.84 ± 2.30	<0.001
FLUTS Voiding	1.67 ± 1.71	1.60 ± 1.77	0.798
FLUTS Incontinence	11.49 ± 3.08	2.37 ± 2.82	<0.001
STAI (total)	50.65 ± 8.29	45.77 ± 7.27	<0.001

Regarding the postoperative results according to the type of incontinence, patients with SUI had better PGI-I and PGI-S scores compared to patients with MUI (1.3 ± 0.7 vs. 1.9 ± 1.1, *P *= 0.033, and 1.1 ± 0.5 vs. 1.7 ± 0.7, *P *= 0.004, respectively). Regarding the postoperative results according the severity of incontinence, there was no statistical difference in PGI-I and PGI-S scores between patients with moderate and severe SUI.

**Figure 1:**
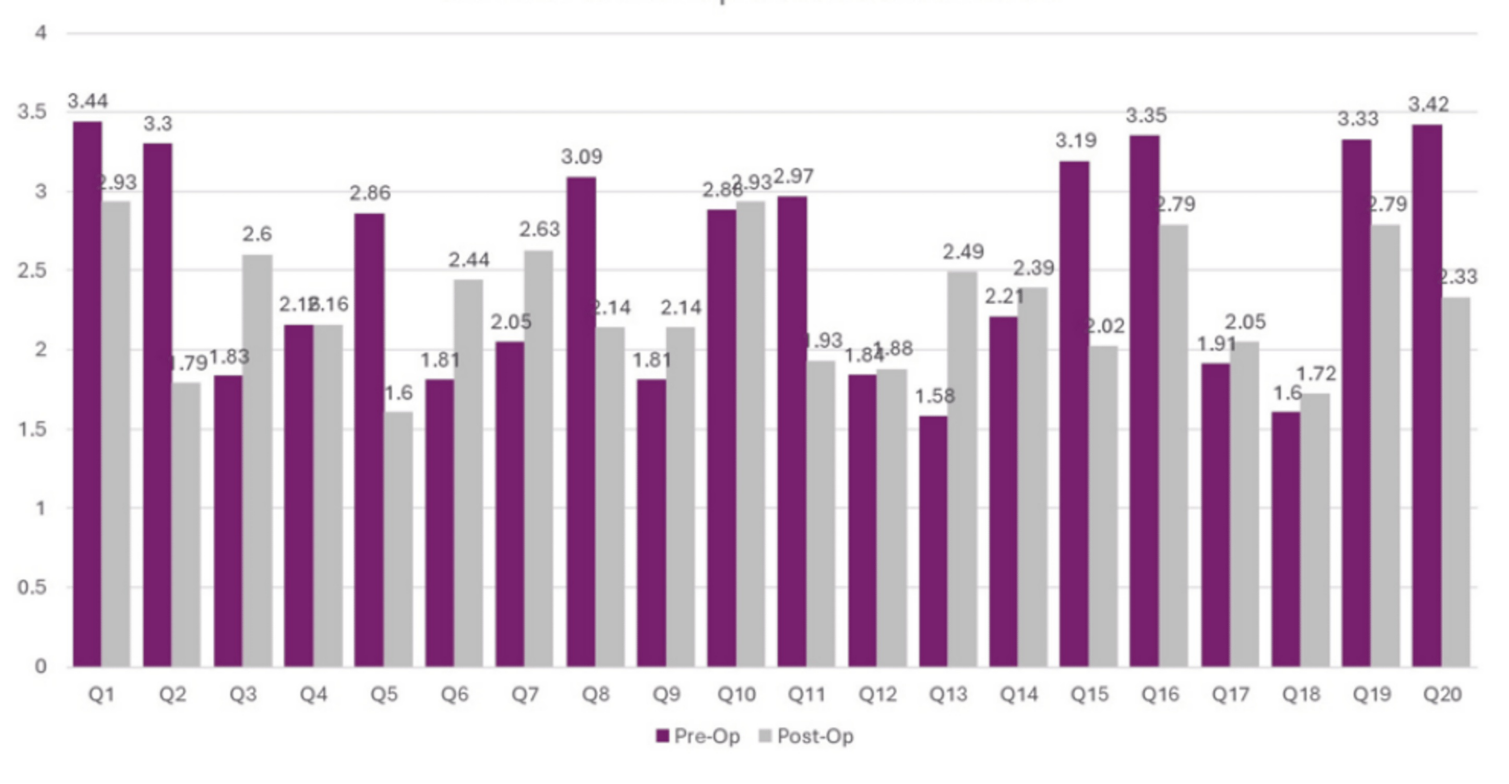
STAI: Pre- vs. postoperative scores. STAI, State-Trait Anxiety Inventory

There was a statistically significant reduction in STAI (total) after surgery (50.6 ± 8.3 vs. 45.8 ± 7.3, *P *< 0.001). The pre- and postoperative STAI subscales (State and Trait) are depicted in Figure 1.

According to the type of incontinence, there was no difference in patients with SUI compared to patients with MUI both in pre-operative (50.9 ± 8.2 vs. 51.2 ± 8.9, *P *= 0.814) and postoperative STAI scores (45.4 ± 6.9 vs. 47.1 ± 8.7, *P *= 0.515).

Preoperatively, patients with moderate SUI had a lower mean STAI (total) score than those with severe SUI (47.1 ± 6.4 vs. 51.9 ± 8.6, *P* = 0.099). Postoperatively, the mean STAI score remained lower in the moderate SUI group compared to the severe SUI group (43.2 ± 8.7 vs. 46.6 ± 8.6, *P* = 0.174). However, there were more patients with moderate SUI who had normal STAI score (<41) compared to the group of patients with severe SUI (*P *= 0.011).

## Discussion

This prospective study was designed to assess the impact of SIMS surgery on anxiety symptoms in women diagnosed with moderate to severe SUI. Utilizing the STAI, we observed a statistically significant reduction in anxiety levels three months postoperatively. These findings suggest improvements in continence following SIMS were associated with meaningful psychological benefits.

Our central hypothesis posited that effective surgical management of SUI with SIMS would lead to a statistically significant reduction in anxiety symptoms, with the most notable improvements observed in state anxiety. Given its association with specific, situational stressors, such as the fear of urine leakage or social embarrassment, state anxiety was expected to show greater responsiveness to symptom resolution. In contrast, we anticipated that trait anxiety, reflecting a more stable personality dimension, might show less pronounced change, particularly in individuals with high baseline levels. The design of the study permitted us to investigate whether reductions in anxiety varied according to baseline severity of SUI and whether preoperative trait anxiety predicted the extent of emotional improvement postoperatively. By addressing these psychological dimensions, the study sought to generate a more comprehensive understanding of the emotional benefits of surgical intervention in women with SUI (with STAI-State and STAI-Trait analyzed separately).

The observed improvement in anxiety is consistent with previous research highlighting the psychological toll of SUI. Involuntary urine leakage is known to extend beyond physical discomfort, often eliciting feelings of shame, fear of public embarrassment, social withdrawal, and avoidance of sexual intimacy [1-3]. These psychosocial consequences frequently manifest as heightened state anxiety-a transient, situation-dependent form of anxiety that is particularly reactive to stressors such as fear of leakage in public environments. Our results indicate that successful surgical correction of SUI is associated with mitigation of this situational psychological distress.

The mean STAI (total) declined significantly from 50.6 ± 8.3 at baseline to 45.8 ± 7.3 postoperatively (*P* < 0.001), reflecting a meaningful improvement in overall mental health. Although the average postoperative score remained marginally above the conventional threshold for *normal* anxiety (<41), a larger proportion of women-particularly those with moderate SUI-achieved normal anxiety scores following surgery. This suggests that, beyond continence restoration, SIMS may facilitate emotional recovery and enhance psychological resilience in affected women.

Interestingly, while patients with moderate SUI exhibited lower mean STAI (total) both before and after surgery compared to those with severe SUI, the difference did not reach statistical significance. However, the proportion of women achieving normal anxiety scores postoperatively was significantly higher in the moderate SUI group. This finding supports the hypothesis that baseline severity of incontinence may influence psychological outcomes, as severe SUI may contribute to more persistent anxiety, entrenched behavioral adaptations (e.g., hypervigilance or avoidance), or even coexisting depressive symptoms that may not fully resolve with surgical intervention alone [4,5].

Notably, both state and trait anxiety improved following surgery; however, the effect was more pronounced among women with moderate SUI. A significantly higher proportion of these patients reached normal anxiety levels after the intervention, compared to those with severe SUI. Although women with more severe symptoms experienced a substantial reduction in anxiety, their postoperative scores were less likely to fall within the normative range. This pattern suggests that baseline severity of SUI may influence not only the psychological burden before treatment but also the degree of emotional recovery afterward, highlighting the importance of individualized perioperative care strategies.

The absence of significant differences in anxiety outcomes between patients with pure SUI and those with mixed urinary incontinence (MUI) indicates that SIMS can provide emotional benefits even in the presence of overlapping symptoms. Nevertheless, the slightly lower satisfaction scores reported by women with MUI may reflect the persistence of urgency-related symptoms, which could temper overall perceptions of improvement. These observations further support the integration of psychological evaluation and follow-up as standard components of the care pathway for women undergoing continence surgery.

These findings align with prior reports that stress-predominant MUI may experience psychological benefit when the stress component is addressed, though residual urgency can modestly reduce satisfaction [6]. Trials testing adjunct therapies for urgency could clarify whether additional symptom control further improves emotional recovery in this subgroup.

In recent years, SIMS have emerged as a minimally invasive alternative to conventional MUS techniques. Unlike retropubic or transobturator approaches, SIMS involve a single vaginal incision and fixation into the obturator internus fascia, resulting in reduced tissue dissection, shorter operative time, and decreased postoperative discomfort [15]. Multiple randomized controlled trials and meta-analyses have demonstrated comparable efficacy between SIMS and traditional slings in carefully selected patients, particularly those with isolated SUI and sufficient urethral mobility [17,24].

SIMS are associated with high rates of both objective and subjective cure, minimal intraoperative complications, and rapid recovery, making them an attractive option for patients seeking low-risk surgical solutions with minimal downtime. Nonetheless, while considerable attention has been paid to their physical outcomes, considerably less is known about their impact on emotional and psychological well-being. Surgical success is typically assessed through parameters such as pad usage, frequency of leakage, or urodynamic findings-metrics that fail to capture the broader psychosocial ramifications of the condition or its treatment [24].

From a clinical standpoint, our findings underscore the value of SIMS not only for its established benefits-such as minimal invasiveness, shorter operative duration, reduced postoperative discomfort, and high continence rates-but also for its potential to improve mental health outcomes. The procedure appears particularly well-suited for women experiencing significant psychological burden from incontinence and who prefer a low-risk surgical option. These results support the integration of psychological screening and follow-up into the standard care pathway for women undergoing incontinence surgery. The STAI proved to be both feasible and sensitive in this setting, enabling assessment of both transient (state) and enduring (trait) anxiety.

Our study further contributes to the growing body of evidence advocating for the use of PROMs as essential indicators of surgical success. Traditional evaluation metrics, such as anatomical restoration or urodynamic parameters, often fail to reflect the patient’s subjective experience and psychosocial well-being [7]. Incorporating validated tools like STAI and ICIQ provides a more comprehensive and patient-centered assessment of treatment efficacy. This approach aligns with recent recommendations from the International Urogynecological Association (IUGA) and the International Continence Society (ICS), which emphasize the inclusion of mental health, sexual function, and QOL outcomes in pelvic floor disorder research [8]. We also used the Greek-validated STAI-Y, acknowledging potential cultural influences on anxiety expression in our population.

A particularly noteworthy aspect of our findings is the relatively rapid onset of psychological improvement, observed within three months post-surgery. This suggests that relief from leakage-related distress and restoration of bodily control may lead to early emotional recovery. However, further longitudinal studies are needed to assess the durability of these benefits and to determine whether adjunct psychological support is necessary for sustained improvement, especially in patients with preexisting psychiatric comorbidities. Individuals with elevated baseline trait anxiety may require more comprehensive, multidisciplinary care involving both urogynecological and psychological support services.

Ultimately, this research underscores the importance of evaluating surgical outcomes not solely on the basis of objective continence measures or urodynamic parameters, but also in terms of psychosocial recovery and mental well-being. Our findings support the integration of routine psychological assessment and follow-up in the perioperative care of women undergoing SUI surgery. Moreover, they highlight the broader therapeutic potential of SIMS procedures, not only in restoring continence but also in facilitating emotional and psychological rehabilitation.

Clinical implications

We recommend brief preoperative STAI screening to identify patients with high trait anxiety, provide perioperative counseling or referral when indicated, and routinely track STAI and ICIQ as PROMs to guide quality metrics and value-based care.

This study advocates for a more holistic approach to urogynecological care, where surgical success is defined not only by continence outcomes but also by improvements in mental health and QOL. Future larger-scale studies with extended follow-up and inclusion of control groups are needed to further validate these findings and explore long-term psychological trajectories following SIMS intervention.

Despite the strengths of our study-including its prospective design, use of validated instruments, and real-world clinical context-several limitations should be acknowledged. The sample size, although sufficient to detect statistically significant changes in primary outcomes, limits the power of subgroup analyses. Larger, multicenter studies would enable more robust comparisons between patient subgroups, such as those with different SUI severities or coexisting MUI. Additionally, our exclusion of women with prior incontinence surgery or ongoing psychiatric treatment may have introduced selection bias, favoring a psychologically healthier cohort. Moreover, although STAI is a well-established tool, it remains a self-reported measure and may be subject to response bias. Finally, the absence of a non-surgical control group restricts our ability to conclusively attribute anxiety improvements to the surgical intervention alone, as spontaneous fluctuations or placebo effects cannot be entirely ruled out.

Future research should aim to replicate these findings in larger, more diverse populations and compare SIMS with alternative treatment modalities, including conservative therapies and other surgical approaches such as traditional mid-urethral slings. In addition, qualitative studies exploring women's lived experiences before and after surgery could offer deeper insight into the mechanisms underlying emotional recovery. Extended follow-up periods and the incorporation of structured psychiatric evaluations would also provide a more complete picture of the psychological trajectory following incontinence surgery.

## Conclusions

This prospective study underscores the dual therapeutic value of SIMS surgery in women with moderate to severe SUI, demonstrating high continence rates and anxiety reductions at three months. Using the STAI, we identified a statistically and clinically meaningful decline in anxiety scores at three months postoperatively. These findings suggest that addressing the physical burden of SUI was associated with psychological relief, particularly by alleviating situational stressors such as fear of leakage and public embarrassment. Clinical implications: brief preoperative STAI screening may identify patients who benefit from perioperative counseling/referral, and routine tracking of STAI/ICIQ as PROMs could support quality improvement and value-based care. Future multicenter studies with longer follow-up and control groups should confirm durability and mechanisms of benefit.
