# Correction: Anxiety Symptoms Before and After Single-Incision Mini-Sling Surgery in Women With Stress Urinary Incontinence: A Prospective Study

**DOI:** 10.7759/cureus.c383

**Published:** 2025-11-25

**Authors:** Drivakou Despoina, Theodoulidis Iakovos, Roussos Nikolaos, Tsiapakidou Sofia, Pantazis Konstadinos, Dinas Konstantinos, Mikos Themistoklis, Mary H Kosmidis

**Affiliations:** 1 Urogynecology Unit, 2nd Department of Obstetrics and Gynecology, Aristotle University of Thessaloniki, Hippokration General Hospital, Thessaloniki, GRC; 2 Urogynecology Unit, 1st Department of Obstetrics and Gynecology, Papageorgiou Hospital, Thessaloniki, GRC; 3 Urogynecology Unit, 1st Department of Obstetrics and Gynecology, Aristotle University of Thessaloniki, Papageorgiou General Hospital, Thessaloniki, GRC; 4 School of Psychology, Aristotle University of Thessaloniki, Thessaloniki, GRC

This article has been corrected to include authors in author positions 2-8 as they were originally omitted by the submitting author. All authors have agreed to these changes.

